# Redox Mechanisms in Regulation of Adipocyte Differentiation: Beyond a General Stress Response

**DOI:** 10.3390/cells1040976

**Published:** 2012-11-05

**Authors:** Guei-Sheung Liu, Elsa C. Chan, Masayoshi Higuchi, Gregory J. Dusting, Fan Jiang

**Affiliations:** 1 Centre for Eye Research Australia, University of Melbourne, Victoria 3002, Australia; Email: guei-sheung.liu@unimelb.edu.au (G.-S.L.); elsa.chan@unimelb.edu.au (E.C.C.); mhiguchi@unimelb.edu.au (M.H.); g.dusting@unimelb.edu.au (G.J.D.); 2 Key Laboratory of Cardiovascular Remodeling and Function Research, Qilu Hospital, Shandong University, Jinan 250-012, Shandong, China

**Keywords:** adipocyte, adipogenesis, differentiation, obesity, oxidative stress, reactive oxygen species, redox regulation

## Abstract

In this review, we summarize advances in our understanding of redox-sensitive mechanisms that regulate adipogenesis. Current evidence indicates that reactive oxygen species may act to promote both the initiation of adipocyte lineage commitment of precursor or stem cells, and the terminal differentiation of preadipocytes to mature adipose cells. These can involve redox regulation of pathways mediated by receptor tyrosine kinases, peroxisome proliferator-activated receptor γ (PPARγ), PPARγ coactivator 1α (PGC-1α), AMP-activated protein kinase (AMPK), and CCAAT/enhancer binding protein β (C/EBPβ). However, the precise roles of ROS in adipogenesis *in vivo* remain controversial. More studies are needed to delineate the roles of reactive oxygen species and redox signaling mechanisms, which could be either positive or negative, in the pathogenesis of obesity and related metabolic disorders.

## 1. Introduction

Adipogenesis is a process by which new adipocytes formed from mesenchymal stem cells or other precursor cells [1,2]. Expansion of the white adipose tissue results in development of obesity, which has significant contributions to hyperglycemia, hyperlipidemia, insulin resistance, chronic inflammation, type 2 diabetes and atherosclerosis [1,2]. Adipocyte differentiation and maturation is a complex developmental process involving a highly orchestrated program of gene expression, and understanding of the molecular mechanisms underlying adipogenesis is important for discovery of new targets to treat obesity-related disorders [3]. Excessive production of reactive oxygen species (ROS) induces oxidative stress in cells. This may lead to cellular damage caused by oxidative modification of lipids, proteins, and DNA [4]. However, it is becoming clear that a non-toxic level of ROS may be involved in transducing intracellular signals and thereby regulating fundamental cell behaviors such as proliferation, differentiation and survival [5,6,7]. Current evidence indicates that ROS may be involved in promoting both of initiation of adipocyte lineage commitment of precursor or stem cells, and the terminal differentiation of preadipocytes to mature fat cells. In this mini-review, we summarize advances in our understanding of redox-sensitive mechanisms that regulate adipogenesis.

## 2. ROS and Redox-Dependent Signaling Mechanisms

ROS are oxygen-containing, short-lived molecules that are highly reactive. The most common examples include superoxide, hydrogen peroxide (H_2_O_2_), hydroxyl radical (^·^OH), nitric oxide (NO) and peroxynitrite (ONOO^−^). ROS are generally considered as unwanted by-products produced from aerobic metabolism, for instance the superoxide molecules during mitochondrial oxidative phosphorylation [8]. If ROS are not removed efficiently by endogenous antioxidant systems, such as superoxide dismutase (SOD), catalase and glutathione peroxidase, they may cause cellular oxidative stress and subsequent cellular damages. Oxidative stress is implicated in pathogenesis of many diseases including cardiovascular disease, diabetes and cancer [9,10]. On the other hand, emerging evidence suggests that ROS generated by cells at a physiological (non-toxic) level could act as transmitters in signaling pathways [5,6,7]. Some well-documented cellular signaling proteins that are sensitive to redox regulation include protein tyrosine phosphatases (PTP), certain serine/threonine and tyrosine kinases, and redox-responsive transcription factors (reviewed in [5,6,11,12]). The presence of redox-sensitive sulfhydryl groups in the key cysteine residues in these signaling proteins or their binding partners is essential for their responsiveness to ROS molecules. Oxidation and reduction of the cysteine sulfhydryl group may act as an “on-off” switch for changing structural configuration of the protein and thereby affecting the activity/affinity of the target. Some transcription factors may be activated by ROS, leading to enhanced target gene expressions in the presence of oxidants. In contrast, oxidation of the key cysteine residues in PTP may inactivate the enzyme, resulting in enhanced signaling events downstream of receptor or non-receptor tyrosine kinases. 

**Figure 1:**
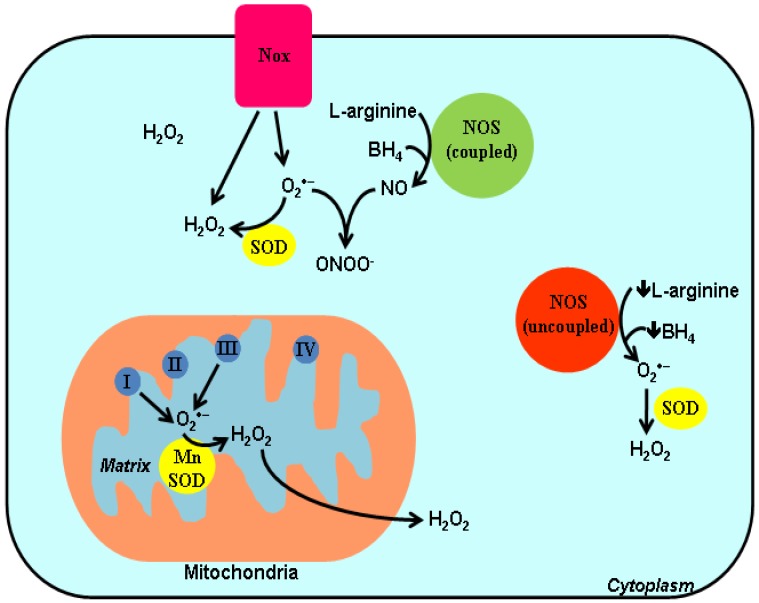
Sources of intracellular ROS generation. Among Nox isoforms (Nox1 to Nox5 and Duox1 and Duox 2), Nox4 is the only isoform that primarily generates hydrogen peroxide (H_2_O_2_) instead of superoxide (O_2_^·^^−^). Superoxide is converted to H_2_O_2_ by endogenous superoxide dismutase (SOD). In mitochondria, superoxide is produced from complexes I and III and is then converted to H_2_O_2_ by manganese SOD (MnSOD). Nitric oxide synthase (NOS) catalyzes the formation of NO from L-arginine; however, NOS can be uncoupled under certain pathological conditions to produce superoxide when the availability of tetrahydrobiopterin (BH_4_) or L-arginine is too low.

ROS can be produced by various enzymes inside the cells (see Figure 1). NADPH oxidase is an important source of intracellular ROS implicated in redox signaling. NADPH oxidase is a multimeric enzyme, and seven isoforms of the catalytic subunit have been identified in mammalian cells, known as Nox1 to Nox5, Duox1 and Duox2. All isoforms have the catalytic domain that allows transport of electron from cytosolic NADPH to generate superoxide, which is then converted to H_2_O_2_ by endogenous SOD [13]. Among these Nox isoforms, Nox4 is highly expressed in preadipocytes [14] and may be involved in modulating preadipocyte proliferation and/or differentiation [15,16]. Unlike other Nox proteins, Nox4 can generate ROS under basal conditions in the absence of exogenous stimuli [13]. Interestingly, Nox4 is the only isoform that primarily produces H_2_O_2_ instead of superoxide [13], and this property of Nox4 may be attributable to an extracellular domain called E-loop, which is longer than that of Nox1 or Nox2 [17]. Mutation of a highly conserved histidine in E-loop abolished the ability of Nox4 for spontaneous dismutation of superoxide to H_2_O_2_. It appears that H_2_O_2_ is the likely signal mediator for regulating cell proliferation, differentiation, migration because it is more cell permeable and has a relatively longer half-life in comparison to superoxide [18]. 

Other important sources of intracellular ROS generation include mitochondria and nitric oxide synthase (NOS). Mitochondria are the core machinery for energy production through oxidative phosphorylation [19]. Superoxide can be generated from one electron reduction of molecular oxygen in complex I and complex III and is then converted to H_2_O_2_ by the mitochondrial manganese SOD [8]. There are other enzymatic sources of ROS generation within mitochondria, such as α-ketoglutarate dehydrogenase [20] located in the matrix, and monoamine oxidase localized at the outer membrane [8]. The biological roles of mitochondria-mediated redox signaling have been extensively reviewed by others [21]. NOS catalyzes the formation of NO from L-arginine via two successive oxygenation steps involving electron transfer process between the C-terminal flavin-containing reductase domain and N-terminal heme-containing oxygenase domain [22]. Under certain pathological conditions, the enzymatic activity of NOS can be uncoupled to produce superoxide rather than NO. For example, when the concentration of NOS cofactor tetrahydrobiopterin or substrate L-arginine is too low [22]. This oxidation of tetrahydrobiopterin facilitates superoxide generation from endothelial cells [23]. The oxidized form of tetrahydrobiopterin is unable to donate electron to synthesize NO, while ONOO^−^ is formed from concomitant generation of superoxide and NO by uncoupled NOS. 

## 3. Molecular Mechanisms Governing Adipocyte Differentiation

Preadipocytes are thought to be derived from mesenchymal stem cells, which are capable of differentiating into multiple lineages including adipocytes, osteoblasts, chondrocytes, and myoblasts [24,25]. Adipogenic process occurs via two components: commitment of mesenchymal stem cells toward preadipocyte fate, and terminal differentiation of preadipocytes into mature adipocytes [25]. Several signaling pathways have been discovered that are sequentially involved in regulating adipogenesis [26]. There is evidence that bone morphogenetic proteins (BMP) BMP2 and BMP4 may induce stem cell commitment to adipocytes [27,28]. In contrast, the canonical Wnt signaling has been shown to inhibit adipogenesis by shifting mesenchymal stem cell fate toward the osteoblast lineage [29,30]. Cell cycle arrest of preadipocytes induced by contact inhibition is essential for the initiation of differentiation to adipocytes [31]. These cells in growth arrest state then undergo a process known as mitotic clonal expansion, in which cells re-enter the cell cycle and repeat several rounds of cell division stimulated by adipogenic stimuli such as cAMP elevating agents, glucocorticoids and insulin [25,32]. These processes coincide with the early phase of adipocyte differentiation [1]. Among the transcription factors activated in the early phase of adipocyte differentiation, CCAAT/enhancer binding protein β (C/EBPβ) and C/EBPδ are the major effectors [33]. C/EBPβ activity is regulated by multiple mechanisms including transcription, phosphorylation and acetylation [25]. C/EBPδ expression level shows a surging response in the early phase and almost disappears in the late phase [1]. cAMP upregulates C/EBPβ via cAMP response element-binding protein (CREB) and Krüppel-like factor 4 (KLF4) [34,35]. Another mediator of C/EBPβ transcription is Krox20, which is rapidly induced by mitogens and, together with KLF4, cooperatively transactivates the C/EBPβ promoter in 3T3-L1 [34,36]. Signal transducer and activator of transcription 3 (STAT3), which is downstream of the Janus kinase 2 (JAK2), regulates C/EBPβ transcription by binding to the distal region of C/EBPβ promoter [37]. Moreover, C/EBPβ needs to be sequentially phosphorylated by mitogen-activated protein kinases (MAPK) and glycogen synthase kinase (GSK)-3β to acquire DNA binding activity [38]. Glucocorticoid, an essential adipogenic agent, enhances C/EBPδ expression [39] and promotes C/EBPβ acetylation, leading to potentiated C/EBP-dependent adipogenic differentiation [40]. Terminal differentiation of adipocytes is primarily regulated by transcription factors C/EBPα and peroxisome proliferator-activated receptor (PPAR)γ, which program gene expressions required for mature adipocytes [33,41]. C/EBPβ and C/EBPδ, along with glucocorticoid receptor, STAT5, and retinoid X receptor (RXR), stimulate expression of PPARγ and C/EBPα [25,33,42]. Subsequently, PPARγ and C/EBPα cooperate to induce expression of adipocyte specific genes such as FABP4, GLUT4, adiponectin, PEPCK, CD36, LPL, AGPAT2, PLIN1, and LEP [43,44,45,46].

PPARγ is the master regulator of terminal differentiation and gene expression during adipogenesis, and many adipogenic signaling pathways target PPARγ activity. Insulin induces phosphorylation of Akt, which then activates mammalian target of rapamycin complex 1 (mTORC1) via inhibition of TSC1/2, leading to activation of SREBP1c, an adipogenic transcription factor which regulates fatty acid synthase, lipoprotein lipase and PPARγ expression [47,48,49,50,51]. Akt also enhances PPARγ expression via nuclear exclusion of Foxo1, which inhibits PPARγ activity [52]. In addition, KLF5, which is upregulated by C/EBPβ and C/EBPδ, activates PPARγ2 promoter and induces 3T3-L1 adipogenic differentiation without hormonal stimulation [53]. Similarly, KLF15 also promotes PPARγ expression and lipid accumulation [54]. In contrast, KLF2 functions as a negative regulator of adipocyte differentiation by inhibiting PPARγ expression [55]. Moreover, sirtuin proteins are also involved in modulating lipid and glucose metabolism by suppressing PPARγ activity [56]. For example, Sirt1 may repress PPARγ function by binding to its cofactors [57], while Sirt2 may repress PPARγ transcriptional activity by activating Foxo1 [52,58]. PPARγ coactivator 1α (PGC-1α) is a transcriptional regulator, which may act as a coactivator of PPARγ. It has been well documented that PGC-1α has critical roles in modulating the expression of genes involved in energy metabolism, including mitochondrial biogenesis, glucose uptake, gluconeogenesis, fatty acid oxidation, and adaptive thermogenesis [59,60,61]. Several lines of evidence suggest that activation of PGC-1α favors formation of brown adipose and maintains the specific thermogenic function of brown fat cells [62,63].

## 4. Emerging Evidence of Redox-Dependent Regulation of Adipogenesis

Several lines of studies have suggested that intracellular ROS derived from NADPH oxidase, mitochondria, and NOS may have a significant role in modulating adipocyte differentiation (summarized in Table 1). There is evidence that NADPH oxidase is a major source of ROS in preadipocytes [14,64]. We and others have shown that elevated expression of NADPH oxidase subunits can be observed in adipose tissues in rodent models of obesity and in humans with extreme insulin resistance [64,65,66]. During differentiation of 3T3-L1 preadipocytes, there was a marked increase in ROS production, which was blocked by inhibitors of NADPH oxidase [64]. In consistent with these data, our previous study demonstrated that in human adipose-derived stem cells, agonists-induced adipogenic differentiation was accompanied by increased ROS generation, while scavenging ROS production inhibited the induced adipogenesis process [15]. Moreover, we found that inhibition of NADPH oxidases suppressed adipogenesis in these cells. Of the different Nox isoforms of NADPH oxidase, studies have focused on Nox4, as Nox4 is highly expressed in preadipocytes [9,14]. Kanda *et al.* found that in mesenchymal stem cells, Nox4-produced intracellular ROS enhanced adipogenic differentiation [15]. Similarly, Schröder and coworkers showed that in 3T3-L1 cells, inhibiting Nox4 expression by gene silencing blocked insulin-induced terminal differentiation to adipocytes [16]. These results suggest that Nox4 may have a positive role in promoting the adipogenesis process, probably by facilitating insulin signaling [67]. Despite these *in vitro* data, however, the precise role of Nox4 in adipogenesis needs to be confirmed in other cell types, and its role *in vivo* remains elusive. For example, it was shown that Nox4 deficiency accelerated development of obesity and insulin-resistance in mice [68]. Moreover, the involvement of other Nox isoforms in adipogenesis is currently unclear.

**Table 1:** Intracellular sources of ROS implicated in modulating adipocyte differentiation

Source of ROS	Experimental models	Primary findings	References
NADPH oxidase	3T3-L1 adipocytes	Increased ROS production in accumulated fat contributes to metabolic syndrome.	[64]
NADPH oxidase	3T3-L1 adipocytes, human preadipocytes	Nox4 acts as a switch from insulin-induced proliferation to differentiation by controlling MKP-1 expression, which limits ERK1/2 signaling.	[16]
NADPH oxidase	Mouse MSCs	Increase in the intracellular ROS level via Nox4 mediates adipocyte differentiation through CREB in MSC.	[15]
Mitochondria	3T3-L1 adipocytes	Increase in mitochondrial ROS production caused by inhibition of the electron transport chain (complex I and V) prevented preadipocyte proliferation.	[69]
Mitochondria	Human MSCs	Mitochondrial metabolism and ROS generation are not simply a consequence of differentiation but are a causal factor in promoting adipocyte differentiation.	[70]
NOS	Rat preadipocytes	NO is involved in the positive modulation of preadipocyte differentiation and eNOS rather than iNOS may be the major isoform involved in modulating adipogenesis.	[71]

ROS: reactive oxygen species; MSC: mesenchymal stem cells; NO: nitric oxide; eNOS: endothelial nitric oxide synthase; iNOS: inducible nitric oxide synthase.

The role of mitochondria in adipocyte differentiation is not well understood, but several studies have suggested that ROS produced from dysfunctional mitochondria are associated with altered adipocyte function in diseases such as the metabolic syndrome, diabetes and obesity. Carriere *et al.* studied the role of mitochondrial ROS in regulation of preadipocyte proliferation in 3T3-L1 cells, and demonstrated that an increase in mitochondrial ROS production caused by inhibition of the electron transport chain (complex I and V) prevented preadipocyte proliferation [69]. However, it was observed that in the early phase of adipocyte differentiation of human mesenchymal stem cells, there was an increase in mitochondrial metabolism and ROS generation. Moreover, the authors demonstrated that ROS production from mitochondrial complex III was required for activation of the adipogenic transcriptional cascade via upregulation of C/EBPα and PPARγ [70]. In agreement with these results in human mesenchymal stem cells, we found that inhibiting mitochondrial ROS production with rotenone partially suppressed adipogenic differentiation of human adipose-derived stem cells [71]. Hence, the role of mitochondria-derived ROS in regulating adipogenesis is complex, and appears to be cell type specific. Both endothelial NOS (eNOS) and inducible NOS (iNOS) can be expressed in (pre)adipocytes [72]. With the identification of eNOS and iNOS in adipose cells, there is increasing evidence suggesting that NO (which is also called a reactive nitrogen species or RNS molecule) may have a pivotal regulatory role in adipocyte physiology. For example, it was demonstrated that NO promoted adipogenic differentiation of rat preadipocytes [71]. In vitro differentiation of preadipocytes was accompanied by an increase in iNOS expression, while insulin and angiotensin II increased NO production by preadipocytes [73]. It is likely that NO promotes adipogenesis through activation of the cGMP-PKG pathway [74]. Different clinical studies further demonstrated that the expression levels of NOS and NO production were augmented in the adipose tissue from obese subjects, suggesting that NO might have a role as a modulator of adipogenesis in obesity [73,74,75,76,77]. Nevertheless, the precise role of NOS in adipogenesis needs to be confirmed by further studies. 

Regardless of the specific sources of intracellular ROS production, the close relationship between ROS and adipogenesis has been confirmed by a number of recent studies [78,79,80,81,82,83,84,85]. Different approaches were used in these studies, including pharmacological treatment with antioxidant agents, genetic manipulation of gene expression, and direct measurement of intracellular redox status. Observations form several studies also showed that differentiated adipocytes are metabolically distinct from preadipocytes for adipocytes produce much higher basal levels of intracellular ROS than preadipocytes [16,86,87]. Overall, these results strongly suggest that a more oxidized intracellular environment favors differentiation of progenitor or stem cells into mature adipocytes. Recently, we observed that in human adipose-derived stem cells, overexpression of Nox4 and exogenous application of H_2_O_2_ boosted terminal differentiation into mature adipocytes, further supporting a positive regulatory role of ROS in adipogenesis [88]. Moreover, these *in vitro* data are complemented by the finding that systemic administration of the superoxide scavenging agent tempol in mice prevented the development of obesity [89]. 

Interestingly, an adipocyte phenotype can be induced by various stimuli in non-adipogenic cells via trans-differentiation [90,91,92,93]. For example, treatment with interleukin-17 promoted trans-differentiation of a mouse myoblast cell line into adipocytes [90]. Likewise, it was shown that inhibition of the connexin function with glycyrrhetinic acid in skeletal muscle cells induced a phenotype change into the adipocyte lineage [92]. Overall, these studies suggest that adipocyte trans-differentiation from non-adipogenic cell types is likely to be mediated by a set of transcriptional regulators that are similar to those involved in conventional adipogenesis, such as PPARγ and C/EBP proteins [90,91,92,93]. However, whether cell trans-differentiation into adipocytes is also regulated by redox mechanisms remains to be clarified. 

## 5. Redox-Sensitive Mechanisms Related to Adipogenesis

**Figure 2:**
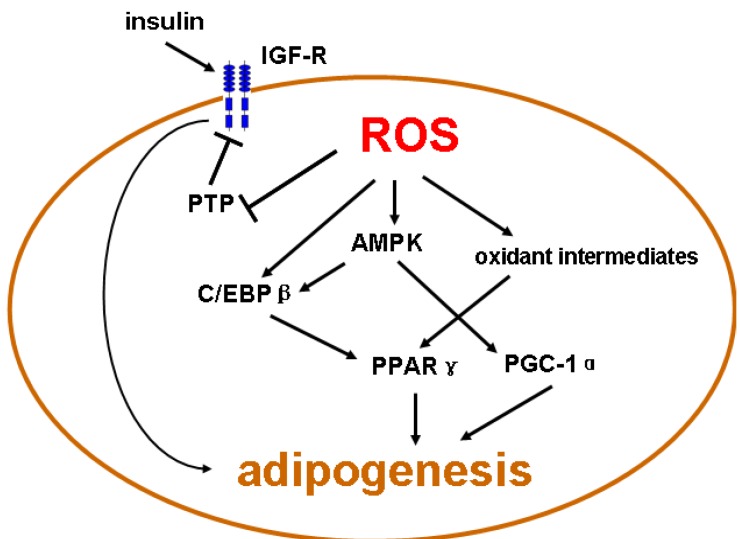
Potential redox-sensitive pathways involved in regulation of adipogenesis. C/EBPβ, CCAAT/enhancer binding protein β; IGF-R, insulin-like growth factor receptor; PTP, protein tyrosine phosphatases; PGC-1α, PPARγ coactivator 1α; AMPK, AMP-activated protein kinase.

How ROS regulate adipogenesis is not completely understood. Different lines of studies have provided some insights into the biological mechanisms underlying redox regulation of adipogenesis (Figure 2). Insulin-like growth factor-1 (IGF-1), an anabolic hormone, has been shown to promote the differentiation of preadipocyte to adipocytes [94]. IGF receptor mediated signaling is responsible for IGF-1 or insulin-induced adipogenesis in 3T3-L1 cells [94]. The IGF receptor is a tyrosine kinase that activates a series of signaling pathways, including the MAPK ERK1/2. It was demonstrated that exposure of 3T3-L1 preadipocytes to an insulin-containing adipogenic cocktail activated ERK1/2, while treatment with ERK pathway inhibitors U0126 or PD98059 significantly attenuated expression of C/EBPα and PPARγ [95]. More than three decades ago, people found that H_2_O_2_ could mimic the metabolic effects of insulin in adipocytes [96,97]. Accumulating evidence now suggests that ROS such as H_2_O_2_ may act as signaling messengers to facilitate biological functions of insulin and IGF-1, primarily by inhibiting endogenous PTP activities [98,99,100]. Likewise, the ERK pathway downstream of receptor tyrosine kinases has been shown to be sensitive to redox regulation in different cells [7]. Indeed we recently demonstrated that Nox4 overexpression in human endothelial cells augmented basal phosphorylation of ERK1/2 [101]. Moreover, ROS have been shown to regulate ERK activation in adipose-derived stem cells [102], which are thought to be precursors of preadipocytes [103]. Current evidence supports that ERK pathway has an important role in the initial lineage commitment of preadipocytes during the differentiation process, but may also have an inhibitory effect on adipocyte maturation [104]. In addition to the ERK pathway, ROS may have positive regulatory actions on other signaling components downstream of the IGF receptor tyrosine kinase that are important to adipogenesis. 

Another adipogenesis-related signaling molecule that might be regulated by redox mechanisms is PPARγ, which is a master regulator of adipogenesis [1,41]. In an epithelial cell line, treatment with an oxidant compound tert-butylhydroperoxide increased the transcriptional activity of PPARγ [105]. In addition, many oxidatively modified biomolecules have been shown to activate PPARγ. For example, UV irradiation induced PPARγ activation in human epithelial like cells, which was mediated by oxidized glycerophosphocholine species with intrinsic PPARγ ligand activity [105]. Similarly, Nagy *et al.* demonstrated that an oxidative product of linoleic acid, hydroxyoctadecadienoic acid, acted as endogenous PPARγ activators and mediated oxidized low density lipoprotein-induced gene expressions in macrophages [106]. The mechanisms of redox-dependent regulation of PPARγ activity remain largely unknown. There is evidence that the Cys-285 located in the LBD domain of PPARγ is redox-sensitive, and the electrophilic nitro-fatty acids can act as partial agonists of PPARγ through covalent binding to Cys-285 [107]. Nitro-fatty acids can be formed by nitration of unsaturated fatty acids under an oxidizing and nitrating condition that could occur during inflammation. So far, however, there is no evidence that PPARγ can be activated by ROS-mediated Cys-285 oxidation, suggesting that oxidative stress-induced PPARγ activation requires an oxidative intermediate. Moreover, although an inhibition of glutathione synthesis has been shown to increase PPARγ expression in 3T3-L1 cells, whether PPARγ can be activated by oxidative stimuli in (pre)adipocytes, and its role in adipogenesis, would require further investigations. 

PGC-1α may also be under redox regulation. In vitro, oxidative stress could induce PGC-1α expression, which was required for the induction of many antioxidant enzymes including glutathione peroxidase and mitochondrial superoxide dismutase (SOD2), indicating that PGC-1α might have an important role in regulating intracellular redox homeostasis [108]. Moreover, PGC-1α has been shown to be a direct substrate of AMP-activated protein kinase (AMPK), and phosphorylation of PGC-1α by AMPK enhanced its transcriptional activity in skeletal muscle cells [109]. Since accumulating evidence has suggested that the functions of AMPK may be tightly regulated by redox status [110], it is plausible that ROS may participate in modulating PGC-1α function, at least indirectly. Supporting these *in vitro* findings, Kang *et al.* carried out an *in vivo* study in rats and demonstrated that endurance exercise increased muscle xanthine oxidase activity and ROS generation. Exercise also upregulated PGC-1α expression, an response that was attenuated by inhibition of xanthine oxidase, suggesting that exercise-activated PGC-1α signaling pathways in skeletal muscle were redox sensitive [111]. Like PPARγ, the nature of redox regulation of PGC-1α signaling and its effects on adipogenic differentiation in precursor or tem cells remain to be elucidated. 

Moreover, redox status may affect the activity of C/EBPβ. It was shown that an oxidative condition further increased the DNA binding of doubly phosphorylated, but not unphosphorylated, C/EBPβ [112]. ROS induced disulfide bond formation between cysteine residues and dimerization of C/EBPβ, leading to further increases in DNA binding activity. This mechanism was supported by the finding that mutation of Cys296 and Cys143 diminished phosphorylation-, oxidation-, and dimerization-dependent DNA binding activity of C/EBPβ [112]. Moreover, it was shown that in 3T3-L1 cells, H_2_O_2_ accelerated hormone-induced adipogenic differentiation with increased expression of PPARγ [113]. H_2_O_2_ also enhanced the mitotic clonal expansion process by promoting cell cycle progression from S to G2/M phase, while treatment with antioxidants resulted in S phase arrest. Oxidant treatment in 3T3-L1 cells resulted in the early appearance of C/EBPβ puncta, which is the characteristic morphological change for enhanced C/EBPβ DNA binding, whereas antioxidant treatment rapidly dispersed the C/EBPβ puncta [113]. All of these results suggest that oxidative stress may facilitate adipogenesis by modulating C/EBPβ-mediated gene expression program. 

## 6. Conclusion Remarks

Redox mechanisms have critical roles in regulating cellular metabolism, and oxidative stress is an important element in metabolic disorders [114,115,116]. Human studies have revealed that there is a correlation between fat accumulation and systemic oxidative stress in obese people [115,116,117,118]. However, our understanding of the reciprocal relationships between obesity and ROS production remains incomplete. Studies have suggested that ROS may be involved in promoting both of the early differentiation and the subsequent maturation of adipose cells [15,70,78,79,80,81,82,83,84,85,88]. However, the role of ROS in adipogenesis *in vivo* is still controversial, and needs more investigation [68,119]. In adults, obesity is mainly caused by hypertrophy of adipocytes with increased lipid accumulation, while de novo adipocyte formation from stem cells might be responsible for continuous turnover of fat cells in the body [2]. Interestingly, emerging evidence has indicated that in hypertrophic obesity, the number of preadipocytes in the subcutaneous adipose tissue capable of undergoing adipogenic differentiation to mature adipocytes is paradoxically reduced, and it is anticipated that restoring the impaired preadipocyte differentiation in the subcutaneous adipose tissue may be a new approach to prevention of ectopic lipid accumulation and resultant insulin resistance [2]. Adding to this complexity is the multi-facet actions of ROS in cell biology. It is likely that ROS may produce disparate effects on adipogenesis depending on the time, intracellular location and intensity of ROS generation. Therefore, we suggest that more carefully designed *in vivo* studies are needed to precisely define the roles that ROS and redox signaling mechanisms are playing, which could be either positive or negative, in obesity and related metabolic disorders.

## References

[1] Lefterova M.I., Lazar M.A. (2009). New developments in adipogenesis. Trends Endocrinol. Metab..

[2] Gustafson B., Gogg S., Hedjazifar S., Jenndahl L., Hammarstedt A., Smith U. (2009). Inflammation and impaired adipogenesis in hypertrophic obesity in man. Am. J. Physiol. Endocrinol. Metab..

[3] Unger R.H., Clark G.O., Scherer P.E., Orci L. (2010). Lipid homeostasis, lipotoxicity and the metabolic syndrome. Biochim. Biophys. Acta.

[4] Halliwell B., Cross C.E. (1994). Oxygen-derived species: Their relation to human disease and environmental stress. Environ. Health Perspect..

[5] Thannickal V., Fanburg B. (2000). Reactive oxygen species in cell signaling. Am. J. Physiol. Lung Cell Mol. Physiol..

[6] Jiang F., Zhang Y., Dusting G. (2011). NADPH oxidase-mediated redox signaling: roles in cellular stress response, stress tolerance, and tissue repair. Pharmacol. Rev..

[7] Chan E.C., Jiang F., Peshavariya H.M., Dusting G.J. (2009). Regulation of cell proliferation by NADPH oxidase-mediated signaling: Potential roles in tissue repair, regenerative medicine and tissue engineering. Pharmacol. Ther..

[8] Finkel T. (2011). Signal transduction by reactive oxygen species. J. Cell Biol..

[9] Bedard K., Krause K.H. (2007). The NOX family of ROS-generating NADPH oxidases: Physiology and pathophysiology. Physiol. Rev..

[10] Rains J.J. (2011). SK Oxidative stress, insulin signaling, and diabetes. Free Radic. Biol. Med..

[11] Abe J., Berk B. (1998). Reactive oxygen species as mediators of signal transduction in cardiovascular disease. Trends Cardiovasc. Med..

[12] Chiarugi P. (2005). PTPs *versus* PTKs: The redox side of the coin. Free Radic. Res..

[13] Martyn K.D., Frederick L.M., Loehneysen K.V., Dinauer M.C., Knaus U.G. (2006). Functional analysis of Nox4 reveals unique characteristics compared to other NADPH oxidases. Cell. Signal..

[14] Mouche S., Mkaddem S.B., Wang W., Katic M., Tseng Y.H., Carnesecchi S., Steger K., Foti M., Meier C.A., Muzzin P. (2007). Reduced expression of the NADPH oxidase NOX4 is a hallmark of adipocyte differentiation. Biochim. Biophys. Acta.

[15] Kanda Y., Hinata T., Kang S.W., Watanabe Y. (2011). Reactive oxygen species mediate adipocyte differentiation in mesenchymal stem cells. Life Sci..

[16] Schroder K., Wandzioch K., Helmcke I., Brandes R.P. (2009). Nox4 acts as a switch between differentiation and proliferation in preadipocytes. Arterioscler. Thromb. Vasc. Biol..

[17] Takac I., Schröder K., Zhang L., Lardy B., Anilkumar N., Lambeth J., Shah A., Morel F., Brandes R. (2011). The E-loop is involved in hydrogen peroxide formation by the NADPH oxidase Nox4. J. Biol. Chem..

[18] Brown D.I., Griendling K.K. (2009). Nox proteins in signal transduction. Free Radic. Biol. Med..

[19] Papa S., Martino P.L., Capitanio G., Gaballo A., Rasmo D.D., Signorile A., Petruzzella V. (2012). The oxidative phosphorylation system in mammalian mitochondria. Adv. Exp. Med. Biol..

[20] Murphy M. (2009). How mitochondria produce reactive oxygen species. Biochem. J..

[21] Collins Y., Chouchani E.T., James A.M., Menger K.E., Cocheme H.M., Murphy M.P. (2012). Mitochondrial redox signalling at a glance. J. Cell Sci..

[22] Vasquez-Vivar J., Kalyanaraman B., Martasek P., Hogg N., Masters B.S., Karoui H., Tordo P., Jr K.A.P. (1998). Superoxide generation by endothelial nitric oxide synthase: The influence of cofactors. Proc. Natl. Acad. Sci. USA.

[23] Kuzkaya N., Weissmann N., Harrison D., Dikalov S. (2003). Interactions of peroxynitrite, tetrahydrobiopterin, ascorbic acid, and thiols: Implications for uncoupling endothelial nitric-oxide synthase. J. Biol. Chem..

[24] Gesta S., Tseng Y.H., Kahn C.R. (2007). Developmental origin of fat: tracking obesity to its source. Cell.

[25] Cristancho A.G., Lazar M.A. (2011). Forming functional fat: A growing understanding of adipocyte differentiation. Nat. Rev. Mol. Cell. Biol..

[26] Rangwala S.M., Lazar M.A. (2000). Transcriptional control of adipogenesis. Annu. Rev. Nutr..

[27] Huang H., Song T.J., Li X., Hu L., He Q., Liu M., Lane M.D., Tang Q.Q. (2009). BMP signaling pathway is required for commitment of C3H10T1/2 pluripotent stem cells to the adipocyte lineage. Proc. Natl. Acad. Sci. USA.

[28] Tang Q.Q., Otto T.C., Lane M.D. (2004). Commitment of C3H10T1/2 pluripotent stem cells to the adipocyte lineage. Proc. Natl. Acad. Sci. USA.

[29] Zhou H., Mak W., Zheng Y., Dunstan C.R., Seibel M.J. (2008). Osteoblasts directly control lineage commitment of mesenchymal progenitor cells through Wnt signaling. J. Biol. Chem..

[30] Bennett C.N., Longo K.A., Wright W.S., Suva L.J., Lane T.F., Hankenson K.D., MacDougald O.A. (2005). Regulation of osteoblastogenesis and bone mass by Wnt10b. Proc. Natl. Acad.Sci. USA.

[31] Shao D., Lazar M.A. (1997). Peroxisome proliferator activated receptor γ, CCAAT/enhancer-binding protein α, and cell cycle status regulate the commitment to adipocyte differentiation. J. Biol. Chem..

[32] Tang Q.Q., Otto T.C., Lane M.D. (2003). CCAAT/enhancer-binding protein β is required for mitotic clonal expansion during adipogenesis. Proc. Natl. Acad. Sci. USA.

[33] Siersbæk R., Nielsen R., Mandrup S. (2012). Transcriptional networks and chromatin remodeling controlling adipogenesis. Trends Endocrinol. Metab..

[34] Birsoy K., Chen Z., Friedman J. (2008). Transcriptional regulation of adipogenesis by KLF4. Cell. Metab..

[35] Zhang J.W., Klemm D.J., Vinson C., Lane M.D. (2004). Role of CREB in transcriptional regulation of CCAAT/enhancer-binding protein β gene during adipogenesis. J. Biol. Chem..

[36] Chen Z., Torrens J.I., Anand A., Spiegelman B.M., Friedman J.M. (2005). Krox20 stimulates adipogenesis via C/EBP[beta]-dependent and-independent mechanisms. Cell. Metab..

[37] Zhang K., Guo W., Yang Y., Wu J. (2011). JAK2/STAT3 pathway is involved in the early stage of adipogenesis through regulating C/EBPβ transcription. J. Biol. Chem..

[38] Tang Q.Q., Grønborg M., Huang H., Kim J.W., Otto T.C., Pandey A., Lane M.D. (2005). Sequential phosphorylation of CCAAT enhancer-binding protein β by MAPK and glycogen synthase kinase 3β is required for adipogenesis. Proc. Natl. Acad. Sci. USA.

[39] Yeh W.C., Cao Z., Classon M., McKnight S.L. (1995). Cascade regulation of terminal adipocyte differentiation by three members of the C/EBP family of leucine zipper proteins. Genes Dev..

[40] Wiper-Bergeron N., Salem H.A., Tomlinson J.J., Wu D., Haché R.J.G. (2007). Glucocorticoid-stimulated preadipocyte differentiation is mediated through acetylation of C/EBPβ by GCN5. Proc. Natl. Acad. Sci. USA.

[41] Farmer S.R. (2006). Transcriptional control of adipocyte formation. Cell. Metab..

[42] Floyd Z.E., Stephens J.M. (2003). STAT5A promotes adipogenesis in nonprecursor cells and associates with the glucocorticoid receptor during adipocyte differentiation. Diabetes.

[43] Lefterova M.I., Zhang Y., Steger D.J., Schupp M., Schug J., Cristancho A., Feng D., Zhuo D., Stoeckert C.J., Liu X.S. (2008). PPARγ and C/EBP factors orchestrate adipocyte biology via adjacent binding on a genome-wide scale. Genes Dev..

[44] Nielsen R., Pedersen T.Å., Hagenbeek D., Moulos P., Siersbæk R., Megens E., Denissov S., Børgesen M., Francoijs K.J., Mandrup S. (2008). Genome-wide profiling of PPARγ: RXR and RNA polymerase II occupancy reveals temporal activation of distinct metabolic pathways and changes in RXR dimer composition during adipogenesis. Genes Dev..

[45] Tontonoz P., Spiegelman B.M. (2008). Fat and beyond: the diverse biology of PPARγ. Annu. Rev. Biochem..

[46] Lowe C.E., O'Rahilly S., Rochford J.J. (2011). Adipogenesis at a glance. J. Cell Sci..

[47] Kim J.B., Spiegelman B.M. (1996). ADD1/SREBP1 promotes adipocyte differentiation and gene expression linked to fatty acid metabolism. Genes Dev..

[48] Bakan I., Laplante M. (2012). Connecting mTORC1 signaling to SREBP-1 activation. Curr. Opin. Lipidol..

[49] Kim J.B., Wright H.M., Wright M., Spiegelman B.M. (1998). ADD1/SREBP1 activates PPARγ through the production of endogenous ligand. Proc. Natl. Acad. Sci. USA.

[50] Moldes M., Boizard M., Liepvre X., Fève B., Dugail I., Pairault J. (1999). Functional antagonism between inhibitor of DNA binding (Id) and adipocyte determination and differentiation factor 1/sterol regulatory element-binding protein-1c (ADD1/SREBP-1c) trans-factors for the regulation of fatty acid synthase promoter in adipocytes. Biochem. J..

[51] Zhang H.H., Huang J., Düvel K., Boback B., Wu S., Squillace R.M., Wu C.L., Manning B.D. (2009). Insulin stimulates adipogenesis through the Akt-TSC2-mTORC1 pathway. PLoS One.

[52] Wang F., Tong Q. (2009). SIRT2 suppresses adipocyte differentiation by deacetylating FOXO1 and enhancing FOXO1's repressive interaction with PPARγ. Mol. Biol. Cell..

[53] Oishi Y., Manabe I., Tobe K., Tsushima K., Shindo T., Fujiu K., Nishimura G., Maemura K., Yamauchi T., Kubota N. (2005). Krüppel-like transcription factor KLF5 is a key regulator of adipocyte differentiation. Cell. Metab..

[54] Mori T., Sakaue H., Iguchi H., Gomi H., Okada Y., Takashima Y., Nakamura K., Nakamura T., Yamauchi T., Kubota N. (2005). Role of Krüppel-like factor 15 (KLF15) in transcriptional regulation of adipogenesis. J. Biol. Chem..

[55] Banerjee S.S., Feinberg M.W., Watanabe M., Gray S., Haspel R.L., Denkinger D.J., Kawahara R., Hauner H., Jain M.K. (2003). The Krüppel-like factor KLF2 inhibits peroxisome proliferator-activated receptor-γ expression and adipogenesis. J. Biol. Chem..

[56] Houtkooper R.H., Pirinen E., Auwerx J. (2012). Sirtuins as regulators of metabolism and healthspan. Nat. Rev. Mol. Cell. Biol..

[57] Picard F., Kurtev M., Chung N., Topark-Ngarm A., Senawong T., De Oliveira R.M., Leid M., McBurney M.W., Guarente L. (2004). Sirt1 promotes fat mobilization in white adipocytes by repressing PPAR-γ. Nature.

[58] Jing E., Gesta S., Kahn C.R. (2007). SIRT2 regulates adipocyte differentiation through FoxO1 acetylation/deacetylation. Cell. Metab..

[59] Liu C., Lin J.D. (2011). PGC-1 coactivators in the control of energy metabolism. Acta Biochim. Biophys. Sin. (Shanghai).

[60] Fernandez-Marcos P.J., Auwerx J. (2011). Regulation of PGC-1alpha, a nodal regulator of mitochondrial biogenesis. Am. J. Clin. Nutr..

[61] Bonen A. (2009). PGC-1alpha-induced improvements in skeletal muscle metabolism and insulin sensitivity. Appl. Physiol. Nutr. Metab..

[62] Huang P.I., Chen Y.C., Chen L.H., Juan C.C., Ku H.H., Wang S.T., Chiou S.H., Chiou G.Y., Chi C.W., Hsu C.C. (2011). PGC-1alpha mediates differentiation of mesenchymal stem cells to brown adipose cells. J. Atheroscler. Thromb..

[63] Spiegelman B.M., Puigserver P., Wu Z. (2000). Regulation of adipogenesis and energy balance by PPARgamma and PGC-1. Int. J. Obes. Relat. Metab. Disord..

[64] Furukawa S., Fujita T., Shimabukuro M., Iwaki M., Yamada Y., Nakajima Y., Nakayama O., Makishima M., Matsuda M., Shimomura I. (2004). Increased oxidative stress in obesity and its impact on metabolic syndrome. J. Clin. Invest..

[65] Park H.S., Jin D.K., Shin S.M., Jang M.K., Longo N., Park J.W., Bae D.S., Bae Y.S. (2005). Impaired generation of reactive oxygen species in leprechaunism through downregulation of Nox4. Diabetes.

[66] Jiang F., Lim H.K., Morris M.J., Prior L., Velkoska E., Wu X., Dusting G.J. (2011). Systemic upregulation of NADPH oxidase in diet-induced obesity in rats. Redox Rep..

[67] Mahadev K., Motoshima H., Wu X., Ruddy J.M., Arnold R.S., Cheng G., Lambeth J.D., Goldstein B.J. (2004). The NAD(P)H oxidase homolog Nox4 modulates insulin-stimulated generation of H_2_O_2_ and plays an integral role in insulin signal transduction. Mol. Cell. Biol..

[68] Li Y., Mouche S., Sajic T., Veyrat-Durebex C., Supale R., Pierroz D., Ferrari S., Negro F., Hasler U., Feraille E. (2012). Deficiency in the NADPH oxidase 4 predisposes towards diet-induced obesity. Int. J. Obes. (Lond).

[69] Carriere A., Fernandez Y., Rigoulet M., Penicaud L., Casteilla L. (2003). Inhibition of preadipocyte proliferation by mitochondrial reactive oxygen species. FEBS Lett..

[70] Tormos K.V., Anso E., Hamanaka R.B., Eisenbart J., Joseph J., Kalyanaraman B., Chandel N.S. (2011). Mitochondrial complex III ROS regulate adipocyte differentiation. Cell. Metab..

[71] Yan H., Aziz E., Shillabeer G., Wong A., Shanghavi D., Kermouni A., Abdel-Hafez M., Lau D.C. (2002). Nitric oxide promotes differentiation of rat white preadipocytes in culture. J. Lipid. Res..

[72] Ribiere C., Jaubert A.M., Gaudiot N., Sabourault D., Marcus M.L., Boucher J.L., Denis-Henriot D., Giudicelli Y. (1996). White adipose tissue nitric oxide synthase: A potential source for NO production. Biochem. Biophys. Res. Commun..

[73] Engeli S., Janke J., Gorzelniak K., Bohnke J., Ghose N., Lindschau C., Luft F.C., Sharma A.M. (2004). Regulation of the nitric oxide system in human adipose tissue. J. Lipid. Res..

[74] Zhang X., Ji J., Yan G., Wu J., Sun X., Shen J., Jiang H., Wang H. (2010). Sildenafil promotes adipogenesis through a PKG pathway. Biochem. Biophys. Res. Commun..

[75] Choi J.W., Pai S.H., Kim S.K., Ito M., Park C.S., Cha Y.N. (2001). Increases in nitric oxide concentrations correlate strongly with body fat in obese humans. Clin. Chem..

[76] Elizalde M., Ryden M., van Harmelen V., Eneroth P., Gyllenhammar H., Holm C., Ramel S., Olund A., Arner P., Andersson K. (2000). Expression of nitric oxide synthases in subcutaneous adipose tissue of nonobese and obese humans. J. Lipid. Res..

[77] Ryden M., Elizalde M., van Harmelen V., Ohlund A., Hoffstedt J., Bringman S., Andersson K. (2001). Increased expression of eNOS protein in omental *versus* subcutaneous adipose tissue in obese human subjects. Int. J. Obes. Relat. Metab. Disord..

[78] Younce C., Kolattukudy P. (2012). MCP-1 induced protein promotes adipogenesis via oxidative stress, endoplasmic reticulum stress and autophagy. Cell Physiol. Biochem..

[79] Liu H., Yang X., Zhang Y., Dighe A., Li X., Cui Q. (2012). Fullerol antagonizes dexamethasone-induced oxidative stress and adipogenesis while enhancing osteogenesis in a cloned bone marrow mesenchymal stem cell. J. Orthop. Res..

[80] Nam W.S., Park K.M., Park J.W. (2012). RNA interference targeting cytosolic NADP(+)-dependent isocitrate dehydrogenase exerts anti-obesity effect *in vitro* and *in vivo*. Biochim. Biophys. Acta.

[81] Hou Y., Xue P., Bai Y., Liu D., Woods C.G., Yarborough K., Fu J., Zhang Q., Sun G., Collins S. (2012). Nuclear factor erythroid-derived factor 2-related factor 2 regulates transcription of CCAAT/enhancer-binding protein beta during adipogenesis. Free Radic. Biol. Med..

[82] Imhoff B.R., Hansen J.M. (2011). Differential redox potential profiles during adipogenesis and osteogenesis. Cell. Mol. Biol. Lett..

[83] Saitoh Y., Xiao L., Mizuno H., Kato S., Aoshima H., Taira H., Kokubo K., Miwa N. (2010). Novel polyhydroxylated fullerene suppresses intracellular oxidative stress together with repression of intracellular lipid accumulation during the differentiation of OP9 preadipocytes into adipocytes. Free Radic. Res..

[84] Samuni Y., Cook J.A., Choudhuri R., Degraff W., Sowers A.L., Krishna M.C., Mitchell J.B. (2010). Inhibition of adipogenesis by Tempol in 3T3-L1 cells. Free Radic. Biol. Med..

[85] Vigilanza P., Aquilano K., Baldelli S., Rotilio G., Ciriolo M.R. (2011). Modulation of intracellular glutathione affects adipogenesis in 3T3-L1 cells. J. Cell Physiol..

[86] Calzadilla P., Sapochnik D., Cosentino S., Diz V., Dicelio L., Calvo J.C., Guerra L.N. (2011). *N*-Acetylcysteine Reduces Markers of Differentiation in 3T3-L1 Adipocytes. Int. J. Mol. Sci..

[87] Imhoff B.R., Hansen J.M. (2010). Extracellular redox environments regulate adipocyte differentiation. Differentiation.

[88] Higuchi M., Dusting G.J., Peshavariya H., Jiang F., Hsiao S.T.-F., Chan E.C., Liu G.-S. (2012). Differentiation of human adipose-derived stem cells into fat involves reactive oxygen species and Forkhead Box O1 mediated upregulation of antioxidant enzymes. Stem. Cells Dev..

[89] Mitchell J.B., Xavier S., DeLuca A.M., Sowers A.L., Cook J.A., Krishna M.C., Hahn S.M., Russo A. (2003). A low molecular weight antioxidant decreases weight and lowers tumor incidence. Free Radic. Biol. Med..

[90] Lee S.J., Lee E.J., Kim S.H., Choi I., Lee D.M., Lee H.J., Yoon D., Chun T. (2011). IL-17A promotes transdifferentiation of mouse myoblast cells (C2C12) into adipocytes by increasing the expression of peroxisome proliferator-activated receptor gamma through CAAT/enhancer binding protein beta signaling. Biotechnol. Lett..

[91] Itoigawa Y., Kishimoto K.N., Okuno H., Sano H., Kaneko K., Itoi E. (2010). Hypoxia induces adipogenic differentitation of myoblastic cell lines. Biochem. Biophys. Res. Commun..

[92] Yamanouchi K., Yada E., Ishiguro N., Nishihara M. (2007). 18alpha-glycyrrhetinic acid induces phenotypic changes of skeletal muscle cells to enter adipogenesis. Cell. Physiol. Biochem..

[93] Liu S., Wang Y., Wang L., Wang N., Li Y., Li H. (2010). Transdifferentiation of fibroblasts into adipocyte-like cells by chicken adipogenic transcription factors. Comp. Biochem. Physiol. A Mol. Integr. Physiol..

[94] Smith P.J., Wise L.S., Berkowitz R., Wan C., Rubin C.S. (1988). Insulin-like growth factor-I is an essential regulator of the differentiation of 3T3-L1 adipocytes. J. Biol. Chem..

[95] Prusty D., Park B.H., Davis K.E., Farmer S.R. (2002). Activation of MEK/ERK signaling promotes adipogenesis by enhancing peroxisome proliferator-activated receptor gamma (PPARgamma ) and C/EBPalpha gene expression during the differentiation of 3T3-L1 preadipocytes. J. Biol. Chem..

[96] Lawrence J.C., Larner J. (1978). Activation of glycogen synthase in rat adipocytes by insulin and glucose involves increased glucose transport and phosphorylation. J. Biol. Chem..

[97] May J.M., de Haen C. (1979). Insulin-stimulated intracellular hydrogen peroxide production in rat epididymal fat cells. J. Biol. Chem..

[98] Meng T.C., Buckley D.A., Galic S., Tiganis T., Tonks N.K. (2004). Regulation of insulin signaling through reversible oxidation of the protein-tyrosine phosphatases TC45 and PTP1B. J. Biol. Chem..

[99] Goldstein B.J., Mahadev K., Wu X. (2005). Redox paradox: insulin action is facilitated by insulin-stimulated reactive oxygen species with multiple potential signaling targets. Diabetes.

[100] Vaquero E.C., Edderkaoui M., Pandol S.J., Gukovsky I., Gukovskaya A.S. (2004). Reactive oxygen species produced by NAD(P)H oxidase inhibit apoptosis in pancreatic cancer cells. J. Biol. Chem..

[101] Datla S.R., Peshavariya H., Dusting G.J., Mahadev K., Goldstein B.J., Jiang F. (2007). Important role of Nox4 type NADPH oxidase in angiogenic responses in human microvascular endothelial cells *in vitro*. Arterioscler. Thromb. Vasc. Biol..

[102] Kim J.H., Park S.H., Park S.G., Choi J.S., Xia Y., Sung J.H. (2011). The pivotal role of reactive oxygen species generation in the hypoxia-induced stimulation of adipose-derived stem cells. Stem. Cells. Dev..

[103] Cawthorn W.P., Scheller E.L., MacDougald O.A. (2012). Adipose tissue stem cells meet preadipocyte commitment: Going back to the future. J. Lipid. Res..

[104] Bost F., Aouadi M., Caron L., Binetruy B. (2005). The role of MAPKs in adipocyte differentiation and obesity. Biochimie.

[105] Zhang Q., Seltmann H., Zouboulis C.C., Konger R.L. (2006). Involvement of PPARgamma in oxidative stress-mediated prostaglandin E(2) production in SZ95 human sebaceous gland cells. J. Invest. Dermatol..

[106] Nagy L., Tontonoz P., Alvarez J.G., Chen H., Evans R.M. (1998). Oxidized LDL regulates macrophage gene expression through ligand activation of PPARgamma. Cell.

[107] Schopfer F.J., Cole M.P., Groeger A.L., Chen C.S., Khoo N.K., Woodcock S.R., Golin-Bisello F., Motanya U.N., Li Y., Zhang J. (2010). Covalent peroxisome proliferator-activated receptor gamma adduction by nitro-fatty acids: Selective ligand activity and anti-diabetic signaling actions. J. Biol. Chem..

[108] St-Pierre J., Drori S., Uldry M., Silvaggi J.M., Rhee J., Jager S., Handschin C., Zheng K., Lin J., Yang W. (2006). Suppression of reactive oxygen species and neurodegeneration by the PGC-1 transcriptional coactivators. Cell.

[109] Jager S., Handschin C., St-Pierre J., Spiegelman B.M. (2007). AMP-activated protein kinase (AMPK) action in skeletal muscle via direct phosphorylation of PGC-1alpha. Proc. Natl. Acad. Sci. USA.

[110] Cardaci S., Filomeni G., Ciriolo M.R. (2012). Redox implications of AMPK-mediated signal transduction beyond energetic clues. J. Cell Sci..

[111] Kang C., O'Moore K.M., Dickman J.R., Ji L.L. (2009). Exercise activation of muscle peroxisome proliferator-activated receptor-gamma coactivator-1alpha signaling is redox sensitive. Free Radic. Biol. Med..

[112] Kim J.W., Tang Q.Q., Li X., Lane M.D. (2007). Effect of phosphorylation and S–S bond-induced dimerization on DNA binding and transcriptional activation by C/EBPbeta. Proc. Natl. Acad. Sci. USA.

[113] Lee H., Lee Y.J., Choi H., Ko E.H., Kim J.W. (2009). Reactive oxygen species facilitate adipocyte differentiation by accelerating mitotic clonal expansion. J. Biol. Chem..

[114] Ando K., Fujita T. (2009). Metabolic syndrome and oxidative stress. Free Radic. Biol. Med..

[115] Keaney J.F., Larson M.G., Vasan R.S., Wilson P.W., Lipinska I., Corey D., Massaro J.M., Sutherland P., Vita J.A., Benjamin E.J. (2003). Obesity and systemic oxidative stress: clinical correlates of oxidative stress in the Framingham Study. Arterioscler. Thromb. Vasc. Biol..

[116] Alfadda A.A., Sallam R.M. (2012). Reactive oxygen species in health and disease. J. Biomed. Biotechnol..

[117] Fujita K., Nishizawa H., Funahashi T., Shimomura I., Shimabukuro M. (2006). Systemic oxidative stress is associated with visceral fat accumulation and the metabolic syndrome. Circ. J..

[118] Vincent H.K., Taylor A.G. (2006). Biomarkers and potential mechanisms of obesity-induced oxidant stress in humans. Int. J. Obes. (Lond).

[119] Galinier A., Carriere A., Fernandez Y., Carpene C., Andre M., Caspar-Bauguil S., Thouvenot J.P., Periquet B., Penicaud L., Casteilla L. (2006). Adipose tissue proadipogenic redox changes in obesity. J. Biol. Chem..

